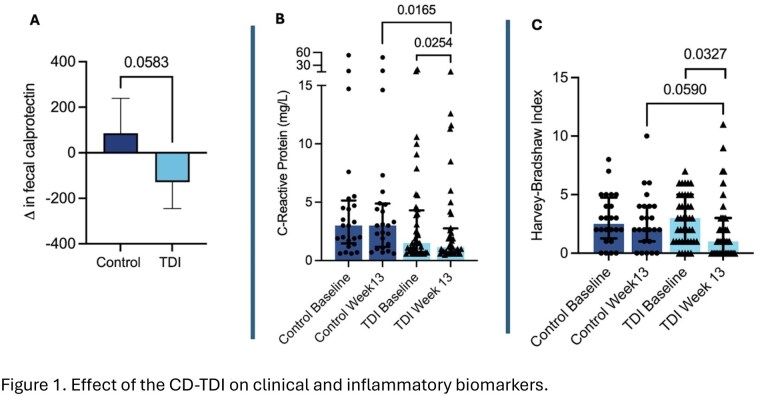# Poster Session I – Poster of Distinction - A87 CROHN’S DISEASE THERAPEUTIC DIET INTERVENTION IMPROVES INTESTINAL PERMEABILITY

**DOI:** 10.1093/jcag/gwaf042.087

**Published:** 2026-02-13

**Authors:** N Haskey, C Ma, L M Taylor, M Yousuf, R A Reimer, S Veniamin, F Peerani, K D McCoy, C Lu, S Ghosh, L A Dieleman, M Raman

**Affiliations:** The University of British Columbia Okanagan Irving K Barber School of Arts and Sciences, Kelowna, BC, Canada; University of Calgary Cumming School of Medicine, Calgary, AB, Canada; LyfeMD, Calgary, AB, Canada; University of Calgary Cumming School of Medicine, Calgary, AB, Canada; University of Calgary Faculty of Kinesiology, Calgary, AB, Canada; University of Alberta Faculty of Medicine & Dentistry, Edmonton, AB, Canada; University of Alberta Faculty of Medicine & Dentistry, Edmonton, AB, Canada; University of Calgary Cumming School of Medicine, Calgary, AB, Canada; University of Calgary Cumming School of Medicine, Calgary, AB, Canada; University College Cork College of Medicine and Health, Cork, County Cork, Ireland; University of Alberta Faculty of Medicine & Dentistry, Edmonton, AB, Canada; University of Calgary Cumming School of Medicine, Calgary, AB, Canada

## Abstract

**Background:**

Defects in the intestinal epithelial barrier are linked to Crohn’s disease (CD) pathogenesis, driving immune activation and chronic inflammation. Circulating zonulin and lipopolysaccharide-binding protein (LBP) are established biomarkers of intestinal permeability (IP), with elevated levels indicating barrier dysfunction. Dietary modulation of these pathways offers a promising strategy to restore mucosal integrity and reduce inflammation. The CD Therapeutic Dietary Intervention (CD-TDI) is a whole-food approach emphasizing polyphenols, β-carotene, soluble fibre, resistant starch, and flavanols, while minimizing ultra-processed foods (UPFs) and additives.

**Aims:**

To evaluate the effect of the CD-TDI as adjunct therapy on biomarkers of IP in individuals with mild-moderate luminal CD.

**Methods:**

In this 13-week, multicenter, RCT, participants were assigned to the CD-TDI (*n*=42) or the habitual diet (control, *n*=24). Serum zonulin and LBP were measured at baseline (BL) and week 13 (Wk13). Linear regression was used to assess associations between the changes in biomarkers of IP and diet adjusting for age, BMI, sex, and disease activity (Harvey-Bradshaw Index [HBI], CRP, fecal calprotectin [FCP]).

**Results:**

Paired serum samples were available from 64 participants (40 CD-TDI, 24 controls; mean age of 47±15 years; 53% female; BMI 28±5.5 kg/m^2^). At BL, disease activity was mild-moderate (HBI 3±2; FCP 365±320 mcg/g) and 34% were on biologics, 8% corticosteroids, 23% 5-ASA, and 17% immunosuppressants or combination therapy. The CD-TDI group demonstrated improved clinical disease activity and intestinal inflammation compared to controls (Figure 1A-C), supporting the rationale to evaluate its effect on IP.

Within the CD-TDI group, significant reductions were observed in zonulin (from 259→145 ng/mL, p = 0.001) and LBP (from 20→17 µg/mL, p = 0.04) from BL to Wk13, with no change in the control group. Between groups, LBP remained significantly lower in the CD-TDI group (p = 0.01). Adjusted analyses confirmed that the CD-TDI group had a 69 ng/mL greater reduction in zonulin **(**β= –69.4 (95% CI:–131.2 to–7.6), p = 0.03) and a 3.2 µg/mL greater reduction in LBP (β=–3.17, p = 0.03) relative to the control group. Changes in zonulin and LBP closely paralleled changes in systemic inflammation (ΔCRP), with exploratory analyses suggesting that higher UPF exposure (e.g., added sugars, cured meats) was associated with systemic inflammation, whereas plant-rich, nutrient-dense dietary components (e.g., dark-green vegetables, vitamin B6) trended toward a protective effect on barrier function.

**Conclusions:**

These findings suggest that the beneficial effects of the CD-TDI on IP may be driven by nutrient-dense, minimally processed dietary patterns that enhance mucosal integrity and dampen systemic inflammation.

**Funding Agencies:**

CCFA, Health Research BC Michael Smith Health Professional Investigator Award, TRIANGLE